# DSM265 at 400 Milligrams Clears Asexual Stage Parasites but Not Mature Gametocytes from the Blood of Healthy Subjects Experimentally Infected with *Plasmodium falciparum*

**DOI:** 10.1128/AAC.01837-18

**Published:** 2019-03-27

**Authors:** Katharine A. Collins, Thomas Rückle, Suzanne Elliott, Louise Marquart, Emma Ballard, Stephan Chalon, Paul Griffin, Jörg J. Möhrle, James S. McCarthy

**Affiliations:** a QIMR Berghofer Medical Research Institute, Herston, QLD, Australia; b Medicines for Malaria Venture, Meyrin, Switzerland; c Q-Pharm Pty. Ltd., Herston, QLD, Australia; d Department of Medicine and Infectious Diseases, Mater Hospital and Mater Research, South Brisbane, QLD, Australia; e The University of Queensland, Brisbane, QLD, Australia

**Keywords:** CHMI, DSM265, IBSM, antimalarial agents, blood stage, gametocytes, malaria, transmission, volunteer infection study

## Abstract

DSM265 is a novel antimalarial drug in clinical development that acts as a selective inhibitor of *Plasmodium* dihydroorotate dehydrogenase. In a previous phase 1b study, a single 150-mg dose of DSM265 showed partial efficacy against experimentally induced blood-stage Plasmodium falciparum malaria (IBSM). Pharmacokinetic/pharmacodynamic modeling predicted a human efficacious dose of 340 mg. The primary objectives of the current study were to determine the safety and efficacy of a single oral 400-mg dose of DSM265 against P. falciparum in the IBSM model. Eight healthy participants were inoculated intravenously with 2,800 parasites and treated with DSM265 7 days later. Unexpectedly, one participant did not develop parasitemia during the study. All other participants developed parasitemia, with the complete clearance of asexual parasites occurring following DSM265 treatment. All seven subjects also became gametocytemic. The secondary objectives were to investigate the gametocytocidal and transmission-blocking activity of a second 400-mg dose of DSM265, which was administered 23 days after inoculation. Gametocytes were not cleared by the second dose of DSM265, and transmission-blocking activity could not be determined due to low gametocyte densities. Three DSM265-related adverse events occurred, including a cutaneous rash in one subject on the day of the second DSM265 dose. The results obtained in this study support the prediction of the efficacious dose of DSM265 and provide further evidence that DSM265 is generally safe and well tolerated. In addition, this study confirms preclinical data indicating that DSM265 permits the development and maturation of gametocytes and does not clear mature circulating gametocytes. (This study has been registered at ClinicalTrials.gov under identifier NCT02573857.)

## Abstract (precis)

DSM265 is a novel antimalarial drug in clinical development that acts as a selective inhibitor of *Plasmodium* dihydroorotate dehydrogenase. In a previous phase 1b study, a single 150-mg dose of DSM265 showed partial efficacy against experimentally induced blood-stage Plasmodium falciparum malaria (IBSM).

## INTRODUCTION

Despite advances in malaria prevention and treatment, in the last 5 years global progress in malaria control has stalled (1). Malaria is still responsible for a large burden of morbidity and mortality worldwide, with an estimated 216 million cases and 442,000 deaths occurring in 2016 (1). Achieving the long-term goal of malaria elimination is hampered by many factors, and of particular concern is the development and spread of antimalarial drug resistance (2). The development of new antimalarial treatments with novel mechanisms of action remains a high priority, with several candidates currently under clinical development (reviewed in reference 3). The required properties of new antimalarial molecules have been defined by outlining a number of target candidate profiles (TCPs) and their intended use in targeting different stages of the malaria parasite life cycle (4). Although accepted as an ambitious goal, the ideal antimalarial treatment would be delivered as a single dose, have activity against multiple parasite life cycle stages, and prevent new infection. Ideally, at the time of treating the asexual parasite infection, the drug used would also prevent the development of the transmissible stage of the malaria parasite (gametocytogenesis), clear mature gametocytes, or block transmission to mosquitoes by rendering the gametocytes sterile. (4). As we move toward elimination, drugs with such activity are highly desirable (5). However, at present, the only licensed antimalarial able to clear mature gametocytes is primaquine (0.25 mg/kg of body weight) (6, 7).

DSM265 is a triazolopyrimidine inhibitor of the *Plasmodium* dihydroorotate dehydrogenase (DHODH). DHODH is an enzyme that is essential in *Plasmodium* species for pyrimidine synthesis, as the pyrimidine salvage pathway is absent in these organisms (8). Preclinical studies indicated that DSM265 is highly selective for *Plasmodium* DHODH and has activity against both the blood and liver stages of Plasmodium falciparum (9, 10). *In vitro* studies also indicated that DSM265 lacks activity against early-stage or mature infectious gametocytes but may inhibit oocyst development, thereby potentially providing transmission-blocking activity (10). Four clinical trials investigating the safety and efficacy of DSM265 in humans as either prophylaxis or blood-stage treatment have been performed, but these did not specifically assess the effect of DSM265 on gametocytemia (11–13). In the first-in-human study in healthy participants, single oral doses between 25 and 1,200 mg were safe and well tolerated and demonstrated a long elimination half-life (*t*_1/2_) of approximately 5 days. Using the induced blood-stage malaria (IBSM) model, a single 150-mg dose of DSM265 was effective in clearing asexual P. falciparum parasites, with recrudescent infections developing in all participants and detection of gametocytes being reported after DSM265 treatment (11). In that study, the MIC of DSM265 in blood was estimated to be 1.04 μg/ml, resulting in a predicted single efficacious dose of 340 mg. Two further phase 1b clinical trials demonstrated good prophylactic activity of DSM265, with complete protection being achieved when a single 400-mg dose was administered 1 day prior to sporozoite challenge and partial protection being achieved when it was administered 3 or 7 days prior to challenge (12, 13). In a phase 2a study where the efficacy of DSM265 against acute uncomplicated P. falciparum infection was evaluated in Peru (14), a single dose of DSM265 rapidly cleared most infections. All 11 patients who received a 400-mg dose achieved a PCR-adjusted adequate clinical and parasitological response (ACPR) by day 14, and 8 of 10 patients receiving a 250-mg dose achieved an ACPR by day 14. Over an observation period of 28 days, 1 of 11 patients in the 400-mg-dose cohort experienced recrudescence and 3 of 10 patients in the 250-mg-dose cohort experienced recrudescence. These studies all demonstrate the safety of DSM265 and support the evaluation of the predicted efficacious dose of DSM265.

Therefore, the present study aimed to evaluate the safety, tolerability, pharmacokinetic (PK) profile, and efficacy of a single 400-mg dose of DSM265 in a volunteer infection study where the P. falciparum parasites were intravenously inoculated. Moreover, we investigated the activity of DSM265 against gametocyte development and maturation and also explored the ability of a second 400-mg dose of DSM265 to clear gametocytemia and block transmission to *Anopheles* vector mosquitoes.

## RESULTS

### Study participants.

This study was conducted from 11 November 2015 to 16 December 2015 in eight healthy malaria-naive adults. The majority of participants were Caucasian (7 participants), 5 were male, and the mean age was 24.8 years (Table 1). All eight participants were inoculated intravenously with 2,800 viable P. falciparum-infected red blood cells on day 0. In five participants, blood-stage parasitemia was first detected by 18S ribosomal DNA (rDNA) quantitative PCR (qPCR) on day 4 postinoculation, with a further two participants becoming qPCR positive on day 5. One participant did not develop detectable blood-stage parasitemia at any time during the study. This participant retrospectively reported an undisclosed history of azithromycin usage prior to inoculation. A retrospective assay for azithromycin from a plasma sample collected on day 0 did not reveal measurable drug. In addition, the participant’s blood was shown to support the growth of P. falciparum when used for *in vitro* parasite culture, suggesting that this participant does not have red blood polymorphisms that inhibit parasite growth. All eight participants were treated with an initial 400-mg dose of DSM265 on day 7; the participant that did not develop parasitemia was dosed in order to assess the safety and pharmacokinetics of DSM265. The seven participants who developed blood-stage parasitemia received a second 400-mg dose of DSM265 on day 23 to investigate its activity against mature gametocytes. At the end of the study (day 28), all eight participants received systematic rescue treatment with artemether-lumefantrine and primaquine.

**TABLE 1:** Participant characteristics^*a*^

Characteristic	Value
Mean (SD) age (years)	24.8 (4.0)
No. (%) of patients by sex	
Male	5 (62.5)
Female	3 (37.5
No. (%) of patients by race	
White	7 (87.5)
Asian	1 (12.5)
Mean (SD) BMI (kg/m^2^)	23.4 (2.0)
Mean (SD) ht (cm)	176.5 (14.7)
Mean (SD) wt (kg)	73.1 (11.5)

a Data are for 8 participants. BMI, body mass index.

### Safety.

Overall, the study showed that DSM265 was generally safe and well tolerated. No serious adverse events (SAEs) were reported. A total of 88 adverse events (AEs) which were mostly mild in severity (87.5%) were reported (see Table S1 in the supplemental material), and the majority were deemed related to malaria parasite infection (77.3%). Eleven AEs were moderate in severity, while none were classified as severe. There were no abnormal laboratory or electrocardiogram (ECG) findings that were considered clinically significant. Two participants reported AEs considered to be possibly related to DSM265 treatment. Mild abdominal tenderness was reported for one participant on the day of initial dosing with DSM265; this lasted approximately 3 days, before resolving spontaneously without intervention. One other participant developed a generalized rash and pruritus on the day of the second DSM265 dose, approximately 3 h after dosing. This skin reaction was of moderate severity and resolved after approximately 12 h with cetirizine and promethazine hydrochloride use.

The severity of the malaria symptoms and signs experienced by the participants during the study was generally mild, as assessed by use of a clinical scoring tool. At the time the first dose of DSM265 was administered, 4 of the 7 participants who were parasitemic had no malaria symptoms or signs (clinical score, 0), while the remaining 3 participants each had a score of 3 (Table S2). The peak malaria clinical score recorded during the study was 8; this was recorded for a single participant approximately 36 h after administration of the first dose of DSM265.

### Pharmacokinetic profile of DSM265.

**FIG 1:**
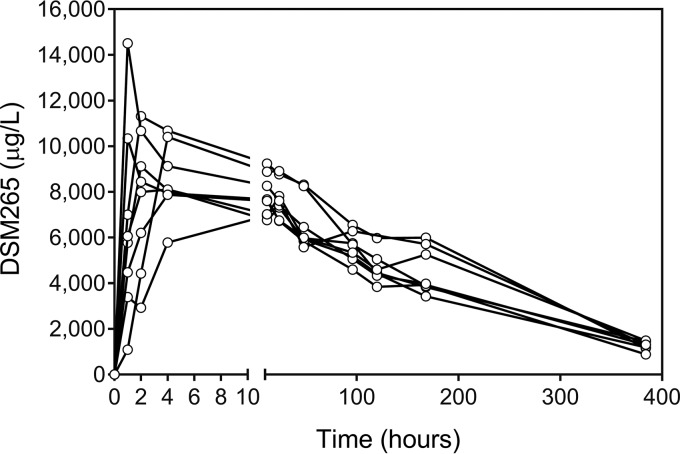
DSM265 plasma concentration-time profiles. Participants received a single dose of DSM265 at 400 mg on day 7. The lines indicate the DSM256 concentration in plasma over time for each participant following DSM265 dosing (*n* = 8).

The plasma pharmacokinetic profile of DSM265 was characterized in all participants from the time of the initial dose of DSM265 until the time of administration of the second dose (Fig. 1). The mean peak DSM265 plasma concentration was 10 μg/ml, which occurred at a median of 2 h postadministration. The area under the concentration-time curve from 0 h extrapolated to infinity (AUC_0–∞_) was estimated to be 1,808 μg·h/ml, and the mean terminal half-life was estimated to be 140.3 h (Table 2).

**TABLE 2:** Noncompartmental pharmacokinetic analysis of DSM265 plasma concentration-time profiles^*a*^

Parameter	Value
*C*_max_ (ng/ml)	9,986 (7,813–14,504)
*t*_max_ (h)	2 (1–24)
AUC_0–∞_ (ng·h/ml)	1,807,590 (1,598,235–2,049,829)
AUC_0–last_ (ng·h/ml)	1,544,339 (1,354,506–1,854,997)
*t*_1/2_ (h)	140 (106–164)

a The data represent the geometric mean (range) for all parameters except *t*_max_, for which the data represent the median (range). Participants (*n* = 8) were treated with a single dose of DSM265 on day 7. The data included in the PK analysis encompass time points from the day of treatment until the day of second dose administration. Abbreviations: AUC_0–last_, the area under the concentration-time curve from 0 h up to the last time point measure; AUC_0–∞_, area under the concentration-time curve from 0 h extrapolated to infinity; *t*_1/2_, elimination half-life; *C*_max_, maximum plasma concentration; *t*_max_, the time point when *C*_max_ was reached; *t*_1/2_, elimination half-life.

### Pharmacodynamic activity of DSM265.

**FIG 2:**
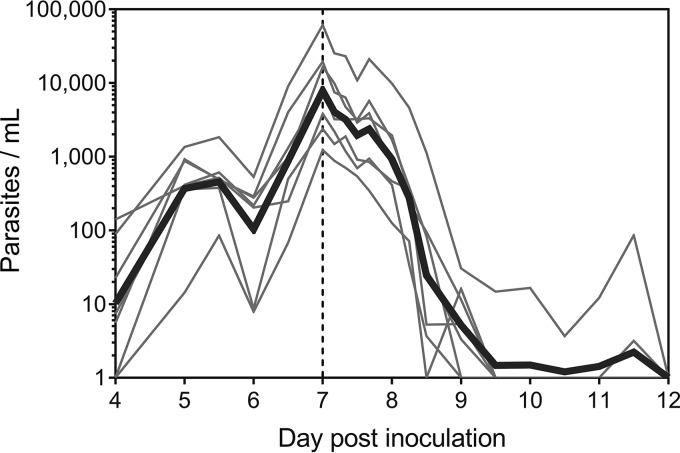
Time course of asexual parasitemia. Participants were inoculated with ∼2,800 viable parasites on day 0, and a single 400-mg dose of DSM265 was administered on day 7 (indicated by the vertical dashed line). Blood-stage parasitemia development and clearance were monitored by 18S rDNA qPCR. Thin lines represent each of the 7 participants who developed parasitemia, and the bold line represents the geometric mean.

Among the seven participants who became parasitemic, the geometric mean parasitemia upon initial dosing with 400 mg DSM265 was 7,851 parasites/ml (95% confidence interval [CI], 2,245 to 27,462 parasites/ml). A reduction in parasitemia was observed within 48 h of DSM265 administration, with complete clearance occurring in all seven participants (Fig. 2). No recrudescent asexual parasitemia developed in any participant. The absence of asexual parasitemia was confirmed by determining that *SBP-1* mRNA transcripts—present only in ring-stage parasites (15, 16)—were absent on day 20. The regression models of the log-linear relationship of parasite decay for the 7 participants who became parasitemic were significant (*P* < 0.001), thereby permitting calculation of the parasite reduction ratio (PRR). The overall cohort-specific ratio of the parasite density at admission and the parasite density 48 h after antimalarial treatment (log_10_ PRR_48_) was 2.78 (95% CI, 2.61 to 2.95), with a corresponding parasite clearance half-life of 5.20 h (95% CI, 4.90 to 5.53 h).

### Effect of DSM265 on gametocytes.

**FIG 3:**
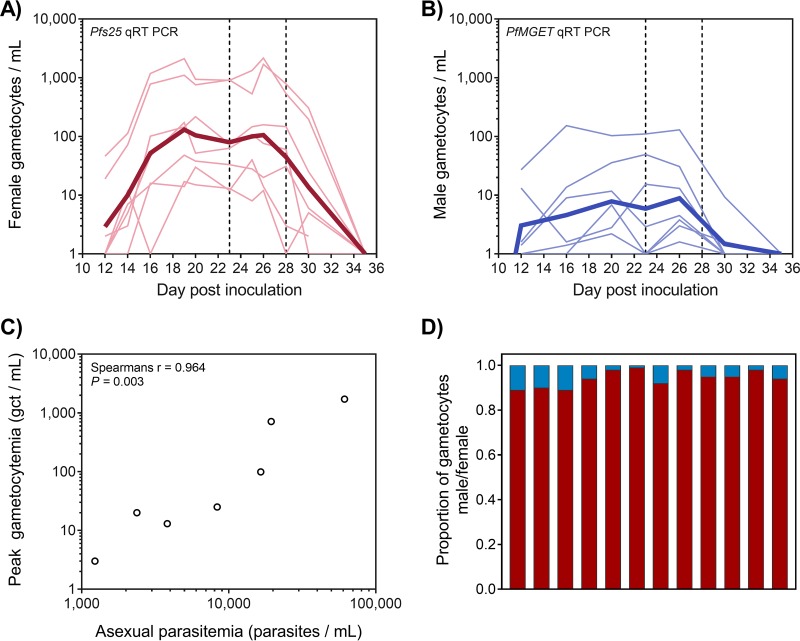
Gametocytemia. (A, B) The gametocyte density was quantified using qRT-PCR assays specific for female gametocytes (*pfs25*) (A) and male gametocytes (*PfMGET*) (B). The first vertical dashed line indicates the day of the second dose of 400 mg DSM265 (day 23), and the second vertical dashed line indicates the end of study primaquine treatment (day 28). Thin lines represent curves for the individual participants, and the bold line is the geometric mean. (C) Comparison of peak gametocytemia (total number of gametocytes [gct] per milliliter measured by 18S rDNA qPCR) to asexual parasitemia at the time of treatment (18S rDNA qPCR). (D) The proportion of male (blue bars) and female (red bars) gametocytes was determined in the 12 samples where male and female gametocyte densities exceeded 100 gametocytes/ml. The samples were from participants R102, R103, and R107 and were taken at multiple time points between day 16 and day 26.

Following the clearance of asexual parasitemia, male and female gametocytes were detected in all seven participants, with low but relatively stable gametocytemia beginning from approximately day 16 after inoculation (9 days after initial DSM265 administration; Fig. 3A and B). The geometric mean level of gametocytemia on day 20 was 105 gametocytes/ml (range, 15 to 937 gametocytes/ml). Peak gametocytemia correlated with asexual parasitemia on the day of DSM265 dosing (day 7), suggesting a relatively consistent gametocyte conversion rate among the participants (Spearman’s *r* = 0.964, *P* = 0.003; Fig. 3C). Male gametocytes were present at lower densities than female gametocytes (Fig. 3A and B). In samples where both male and female gametocyte levels exceeded 100 gametocytes/ml, calculated gametocyte sex ratios were heavily female biased, with a mean of 1 male to 16 female gametocytes (range, 1:8 to 1:63; Fig. 3D). Administration of a second 400-mg dose of DSM265 on day 23 (geometric mean, 80 gametocytes/ml; range, 12 to 915 gametocytes/ml) did not result in a decrease in male or female gametocyte densities. All participants subsequently received systematic rescue treatment with artemether-lumefantrine and a single 45-mg dose of primaquine on day 28, which resulted in clearance of the gametocytes.

### Transmission of parasites to Anopheles stephensi mosquitoes.

To evaluate the transmission-blocking activity of DSM265, mosquito feeding assays were performed during the period of stable gametocytemia, before and after the second 400-mg dose of the drug. Direct skin feeding assays (DFA) were performed on days 20 and 28, while direct membrane feeding assays (DMFA) were performed on days 20, 23, and 28. The mosquitoes fed well on the blood, with a median feeding rate of 100% for the DFA and 97.2% for the DMFA. Mosquito colony health was confirmed by demonstration of low adult mosquito mortality at the time of midgut dissection (median mortality rate, 7.7% for the DFA and 10.3% for the DMFA; Table S3). The rate of mosquito infection was evaluated 7 to 10 days after the feeding assay by assessing the presence of midgut oocysts using 18S rDNA qPCR. Oocysts were not detected in any mosquito midgut either before or after DMS265 dosing. This is likely due to the gametocyte densities at the time of feeding being below the level known to reliably result in transmission to mosquitoes.

## DISCUSSION

This study demonstrated that a single oral dose of 400 mg DSM265 is sufficient to completely clear low-level P. falciparum parasitemia (1,230 to 61,357 parasites/ml on the day of treatment) following experimentally induced blood-stage infection in healthy malaria-naive participants. Pharmacodynamic analysis indicated that DSM265 administration resulted in a modest rate of parasite clearance, with an overall cohort-specific log_10_ PRR_48_ of 2.78 and a corresponding parasite clearance half-life of 5.2 h. This was faster than the clearance rate observed when 150 mg of DSM265 was administered (log_10_ PRR_48_, 1.55; parasite clearance half-life, 9.4 h) and similar to the activity of other long-acting drugs that have been tested in the IBSM model, such as mefloquine (10 mg/kg; log_10_ PRR_48_, 2.34, parasite clearance half-life, 6.2 h) (11) and ferroquine (800 mg; log_10_ PRR_48_, 2.21, parasite clearance half-life, 6.5 h) (17). Importantly, no subjects developed recrudescent parasitemia following administration of a 400-mg dose of DSM265 in this study, which supports the predicted efficacious dose calculation from the preceding study (11). Furthermore, the parasite clearance rate observed in the present IBSM study is similar to that observed when DSM265 at 400 mg was administered to patients with P. falciparum infection in Peru (median log_10_ PRR_48_, 2.9; median parasite clearance half-life, 5.0 h) (14), thereby providing further experimental validation of this system. The pharmacokinetic profile of DSM265 in the current study corresponds closely to results obtained in the other published studies (11–14).

DSM265 was generally safe and well tolerated at the 400-mg dose level, with no SAEs or severe AEs being reported and with only 3 AEs that were considered related to DSM265 treatment (mild abdominal tenderness and moderate skin rash/pruritus) being reported in 2 subjects. The onset of a rash that had some features consistent with a drug reaction (due to the fact that it occurred soon after the second dose of DSM265) raised the possibility of an IgE-mediated immunoallergic reaction. A follow-up comprehensive skin prick test study was performed in this particular subject to investigate if this reaction was due to a hypersensitivity reaction to DSM265. The results of that study will be reported separately. The overall favorable safety profile for DSM265 observed in this study agrees with the safety findings from the previous clinical studies (11–13). In the first-in-human study, 55 participants were administered DSM265 at doses ranging from 25 to 1,200 mg; 15 drug-related adverse events were reported, with the most common being headache, while all other drug-related events were reported only once (11). In the first chemoprophylaxis study, 11 participants were treated with 400 mg DSM265, and only 1 participant experienced an adverse event that was considered related to DSM265 (moderate transient elevation in serum bilirubin) (13). Eighteen participants received 400 mg DSM265 in the second chemoprophylaxis study; nine AEs were considered possibly related to DSM265, and these were mild to moderate headache or gastrointestinal events (12). There were no specific safety concerns reported in the recently completed phase 2a study, in which single doses of DSM265 at 250 to 800 mg were administered to 45 malaria patients in Peru (14). Together, the safety results obtained from the clinical studies performed to date indicate that DSM265 is generally safe and well tolerated when administered orally to human participants.

After the clearance of asexual parasites, gametocytemia was observed in all participants, suggesting that DSM265 at 400 mg does not inhibit gametocytogenesis. Male gametocytes were detected at lower densities than female gametocytes, in accordance with the female-biased sex ratios often seen during natural infections (18, 19). However, the gametocyte sex ratios observed in this study (male/female = 1:16) were more female biased than those normally reported for natural infections (male/female = 1:5) and more female biased than those detected in a previous IBSM study using the same P. falciparum 3D7 parasite line, where transmission to mosquitoes was also investigated (male/female = 1:4) (20). While it is possible that DSM265 treatment may have exerted a differential effect on male and female gametocytes and that this may have contributed to the observed absence of transmission, but this study was not powered to determine this effect. In order to evaluate the gametocytocidal activity of DSM265, an additional 400-mg dose was administered to participants when they were gametocytemic (day 23). In the 5 days following administration of the second dose, the gametocyte numbers remained stable (10 to 1,000 female gametocytes/ml and 1 to 100 male gametocytes/ml), indicating that DSM265 does not clear mature gametocytes. The lack of gametocyte clearance activity by DSM265 agrees with previous *in vitro* studies which indicated that the drug had no activity against either early- or late-stage gametocytes (10) and is consistent with the mechanism of action of DSM265 in blocking pyrimidine nucleotide synthesis, which is downregulated in nonreplicating gametocytes (21). Although gametocyte clearance was not observed, DMS265 at 400 mg (either the first or the second treatment) may have damaged or sterilized the gametocytes, thereby inhibiting oocyst formation, as has been reported from *in vitro* studies (10). However, in this trial, oocysts were not detected in mosquitoes fed either before or after the second dose of DSM265, so it was therefore not possible to determine the effect of DSM265 on transmission. The lack of transmission could also be attributable to the relatively low level of gametocytemia observed in this study (day 20 geometric mean, 105 gametocytes/ml; range, 15 to 937 gametocytes/ml). This is supported by our previous data, where transmission did not occur at gametocyte densities below 850 gametocytes/ml (20), and by reports of the transmissibility of natural infections (22–24). The low level of gametocytemia observed is also in agreement with the previously reported relationship between the asexual parasite biomass at the time of treatment and the resulting gametocytemia, with asexual parasitemia at the time of schizontocidal drug treatment being required to exceed ∼50,000 parasites/ml to result in levels of gametocytemia amenable to onward transmission (>3,000 gametocytes/ml) (20). Enrichment of gametocytes using either density gradient centrifugation or magnetic activated cell sorting prior to performing mosquito feeding assays (20) could help in evaluating transmission to mosquitoes in this model in the future. These assays could enable evaluation of the sterilizing effect of a drug on gametocytes even in the presence of low densities.

The observation that one subject who received the P. falciparum blood-stage inoculum in this study did not develop parasitemia represents a highly unexpected result. It is the first time that a participant has not developed blood-stage parasitemia in this model, with 326 subjects having developed PCR patency after intravenous inoculation with this isolate. With this one failure in 326 challenges, statistical analysis indicates a 95% confidence that the true failure rate does not exceed 1.4%. Although we have not been able to definitively determine the cause of this failure, the possibility of the refractoriness of the participant’s erythrocytes has been excluded. While the possibility of an error in preparation or administration of the inoculum cannot be excluded, syringes for all participants in each cohort were prepared as a single batch, and intravenous injection in this participant took place without mishap. Finally, despite the fact that the participant retrospectively reported an undisclosed history of azithromycin usage prior to inoculation, this drug could not be detected in the plasma sample collected on day 0. However, azithromycin is known to be highly cell associated, and therefore, the possibility of drug-induced inhibition of parasite growth cannot be excluded. Two previous reports also exist of failure to induce experimental malaria parasite infection due to undisclosed suspected quinine consumption (25, 26).

The limitations of this study include the low number of subjects, the use of the P. falciparum 3D7 laboratory parasite strain, and the low levels of gametocytemia. Additionally, in order to fully evaluate the transmission-blocking activity in this model, a control group is required. In this control group, gametocytes should be present in transmissible numbers in participants who either have not received drug treatment or have received a drug, such as piperaquine, that permits viable gametocyte development and subsequent transmission. Drug activity could then be interpreted in the context of expected transmission levels. Without this group, it is not possible to definitively determine the reasons for low or no transmission.

In conclusion, this study aimed to investigate the safety and efficacy of the new antimalarial drug DSM265 when administered as a single oral dose of 400 mg to healthy malaria-naive participants experimentally infected with blood-stage P. falciparum and also evaluate its transmission-blocking activity. A 400-mg dose was shown to be safe and well tolerated and to completely clear asexual parasitemia in all participants without any recrudescence. DSM265 did not prevent the maturation of gametocytes, nor did it clear mature circulating gametocytes. If further studies confirm that DSM265 lacks transmission-blocking activity, it will be important to combine this drug with another agent that both prevents gametocytogenesis and kills mature gametocytes.

The main objective of new drug development is to provide new drugs to treat patients in the event that the current artemisinin-based combination therapies (ACTs) are no longer effective. It is important to note that although extensive Kelch mutations are seen in Southeast Asia, the failure to respond clinically to ACT treatment is largely linked to the failure of the partner drugs: amodiaquine, mefloquine, and piperaquine. Both artemether-lumefantrine and pyronaridine-artesunate appear to maintain activity. A second goal, in addition to providing new options to fight resistance, is to simplify therapy, and a single-encounter therapy would clearly have significant advantages in terms of directly observed therapy. It is important to underline that a molecule such as DSM265 is capable of achieving a single-dose cure at a high dose, but this does not preclude the possibility of its use in combination therapy over multiple days at lower doses.

## MATERIALS AND METHODS

### Study design and participants.

This was a phase 1b, open-label, IBSM clinical trial conducted at Q-Pharm Pty. Ltd. (Brisbane, Australia) between October 2015 and June 2016 aiming to investigate the antimalarial activity of DSM265 against P. falciparum. The second part investigated the antimalarial activity of another investigational drug, OZ439, against P. vivax. The results of the second part will be reported in a separate publication.

Healthy men and women (of nonchildbearing potential) aged 18 to 55 years were eligible for inclusion in the study. Individuals were excluded if they had visited an area where malaria is endemic for a period of longer than 2 weeks in the past 12 months or had received systemic therapy with a drug with potential antimalarial activity in the past 6 weeks. Full inclusion and exclusion criteria for this study are included in the supplemental material. All participants gave written informed consent before being included in the study. This study was approved by the QIMR Berghofer Medical Research Institute Human Research Ethics Committee and was registered at ClinicalTrials.gov (trial identifier NCT02573857).

### Procedures.

A cohort of 8 participants was inoculated on day 0 with P. falciparum-infected human erythrocytes (approximately 2,800 viable parasites) as previously described (11). Parasite growth was monitored by collecting blood samples and performing quantitative PCR (qPCR) targeting the gene encoding P. falciparum 18S rRNA (18S rDNA qPCR) from day 4 after inoculation (27). Blood samples for parasite quantification were collected before inoculation, on day 4, twice daily on days 5 and 6, pretreatment on day 7 and 4, 8, 12, 16, 24, 30, and 36 h posttreatment, twice daily from day 9 to day 11, and every 1 to 3 days until day 35.

Participants received a single oral 400-mg dose of DSM265 after an overnight fast when the protocol-specified threshold for commencement of treatment was reached (parasitemia, ≥5,000 parasites/ml). The threshold was reached on day 7 in this study. DSM265 was supplied as 400 mg powder in a bottle (Bend Research Inc., Bend, OR, USA). The powder was suspended in vehicle (0.1% methylcellulose [Methocel A4M], 0.1% polysorbate 80, 0.005% simethicone, 0.05% ethyl vanillin, 0.5% sucralose) to form a 100-ml suspension for oral administration.

The clearance of parasitemia was measured by qPCR, and gametocytemia was monitored using quantitative reverse transcriptase PCR (qRT-PCR) targeting *Pfs25* mRNA, a transcript preferentially expressed in mature female gametocytes, and *PfMGET* mRNA, a transcript preferentially expressed in mature male gametocytes (28). Interpolation of the transcript number to the gametocyte count was undertaken as previously described (20). To distinguish recrudescent asexual parasitemia from gametocytemia, qRT-PCR was performed to measure P. falciparum *SBP-1* mRNA (15, 16, 20).

The gametocytocidal and transmission-blocking properties of DSM265 were assessed by administering a second single oral dose of 400 mg DSM265 on day 23. The timing of this second dose was based on the appearance of gametocytemia observed during the study. All participants received compulsory rescue treatment with a 3-day course of artemether-lumefantrine (Riamet; Novartis Pharmaceuticals Pty. Ltd., Macquarie Park, Australia) and a single 45-mg dose of primaquine (Primacin; BNM Group, Sydney, Australia) at the end of the study on day 28.

Safety assessments were done at screening and at protocol-specified times. Safety parameters included adverse events reporting, physical examination, vital signs, clinical laboratory evaluation, electrocardiograms (ECGs), and malaria clinical score recording. The malaria clinical score served as a clinical indication of the severity of the induced malaria parasite infection; 14 signs and symptoms commonly associated with malaria were graded using a 3-point scale (0 = absent, 1 = mild, 2 = moderate, 3 = severe), and the values were summed in order to generate an overall score (the maximum possible score is 42).

Blood samples to determine the concentrations of DSM265 were taken before initial DSM265 dosing and at the following time points postdosing: 1, 2, 4, 12, 24, 48, 96, 120, 168, and 384 h (before the second dose). Plasma samples were analyzed by high-performance liquid chromatography-tandem mass spectrometry (HPLC-MS/MS) as previously described (11).

The transmission of parasites from participants to 3- to 7-day-old female Anopheles stephensi mosquitoes was measured using both a direct feeding assay (DFA) and a direct membrane feeding assay (DMFA) on day 20, day 23 (DMFA only), and day 28, as previously described (20). For DFAs, ∼35 mosquitoes were allowed to feed on the participant’s forearm for approximately 15 min. For the DMFA, ∼50 mosquitoes were allowed to feed on a water-jacketed membrane feeder filled with venous blood collected into lithium heparin anticoagulant tubes. At 7 to 9 days after blood feeding, the mosquitoes were dissected to quantify the oocysts in midgut preparations using 18S rDNA qPCR.

### *In vitro* parasite culture.

A venous blood sample was collected in lithium heparin from participant R104 at the end of the study for use in an *in vitro* P. falciparum NF54 parasite culture, and parasite growth in the participants’ blood was compared to parasite growth under normal culture conditions. Participant blood or normal culture blood (Australian Red Cross) was washed and added to different culture flasks at 5% hematocrit and 1% parasitemia in duplicate. Two days later, parasitemia was determined and all flasks were subcultured. Two days later, parasite growth was compared between conditions.

### Azithromycin quantitation in plasma sample.

The concentration of azithromycin was determined by hydrophilic interaction ultraperformance liquid chromatography (HILIC-UPLC) coupled with mass spectrometry (Waters Corporation, Milford, MA, USA). Azithromycin was extracted from serum samples using a methanol-containing labeled internal standard in a 96-well plate. A 1-μl aliquot of the supernatant was injected into the HILIC-UPLC system. Azithromycin was measured against a 7-point calibration curve. The interrun imprecision (percent coefficient of variation) across 3 levels of quality control was <8%.

### Outcomes.

The primary endpoints were the safety, pharmacokinetics, and pharmacodynamics (PD) associated with a single dose of 400 mg DSM265. The PK parameters determined using noncompartmental analysis were the maximum plasma concentration (*C*_max_), the time point when *C*_max_ was reached (*t*_max_), the area under the concentration-time curve from 0 h up to the last time point measure (AUC_0–last_), the area under the concentration-time curve from 0 h extrapolated to infinity (AUC_0–∞_), and the elimination half-life (*t*_1/2_). The PD variables of interest in this study were the parasite reduction ratio (PRR) and the parasite clearance half-life. PRR provides an estimate of the efficacy of an antimalarial treatment and is the ratio of the parasite density between that at admission and that 48 h after antimalarial treatment (expressed as the overall cohort-specific log_10_ PRR_48_). Secondary endpoints were the incidence of transmission of gametocytes to *Anopheles* mosquitoes and the effect of a second dose of 400 mg DSM265 on gametocytemia.

A further objective of this study was to define the PK/PD relationship of DSM265 in P. falciparum parasite clearance. PK/PD modeling was performed using data from the current study in conjunction with data from a previous IBSM study in which subjects were administered 150 mg DSM265 (11). The results of this analysis are the focus of a separate report, which is in preparation.

### Statistical analysis.

The sample size of the current study (*n* = 8) was comparable to that of previous P. falciparum IBSM challenge studies and, based on previously published experience, was considered sufficient for obtaining statistically meaningful data on the effects of DSM265 on malaria parasite kinetics.

Noncompartmental PK analysis was performed using R (version 3.3.1) and RStudio (version 0.99.903) software. The area under the curve was determined using a trapezoidal method up to the last time point measure (AUC_0-last_) and extrapolated to infinity (AUC_0–∞_). The gradient of the elimination phase was determined by log-linear regression, leading to calculation of the elimination half-life (*t*_1/2_). The maximal concentration following DSM265 administration (*C*_max_) and the time that it was reached (*t*_max_) were reported. All parameters were summarized using the geometric mean or median and range.

The PRR and parasite clearance half-life were estimated using the slope of the optimal fit for the log-linear relationship of the parasitemia decay (29). Individual PRR and the corresponding 95% CI were calculated using the slope and corresponding standard error (SE) of the optimal regression model. The cohort PRR and parasite clearance half-life were derived using the weighted mean of the optimal slope for participants with an adequate model fit (*P* < 0.001).

Two *post hoc* analyses were performed. To determine if the level of gametocytemia correlated with the asexual parasitemia on the day of DSM265 dosing, Spearman’s rank correlation coefficient was calculated. Furthermore, in samples where both male and female gametocytes exceeded 100 gametocytes/ml, an estimation of the gametocyte sex ratios was performed.

## Supplementary Material

Supplemental file 1
