# Supplementary material for: DSM265 at 400 Milligrams Clears Asexual Stage Parasites but Not Mature Gametocytes from the Blood of Healthy Subjects Experimentally Infected with Plasmodium falciparum
Source: Antimicrob Agents Chemother. 2019 Mar 27;63(4):e01837-18. doi: 10.1128/AAC.01837-18 (PMC6437518; doi:10.1128/AAC.01837-18)
Supplement: Supplemental file 1 [file AAC.01837-18-s0001.pdf]

Table S1: Incidence and severity of adverse events reported during the study

Adverse event	No. participants	No. events	Severity (No. events)		
			Mild	Moderate	Severe
**Abdominal discomfort**	2	2	2		
Abdominal pain upper	1	1	1		
**Abdominal tenderness***	1	1	1		
Administration site haematoma	1	1	1		
**Arthralgia**	3	3	3		
Arthropod bite	1	1	1		
Cardiac murmur	1	1	1		
Catheter site bruise	3	4	4		
Catheter site pain	2	2	2		
Contusion	1	1	1		
**Decreased appetite**	4	5	5		
**Diaphragmalgia**	1	1		1	
**Disturbance in attention**	1	1		1	
Erythema (relating to mosquito feeding)	3	3	3		
**Fatigue**	4	4	4		
**Headache**	6	19	16	3	
**Hyperhidrosis**	1	1	1		
**Lethargy**	5	7	6	1	
**Malaise**	3	6	6		
**Musculoskeletal pain**	1	3	2	1	
**Myalgia**	3	8	7	1	
**Nausea**	2	2	2		
**Neck pain#**	2	2	1	1	
Petechiae	1	1	1		
Pruritus*	1	1		1	
Rash (relating to intravenous administration of malaria inoculum)	1	1	1		
Rash generalised*	1	1		1	
**Pyrexia**	2	2	2		
Rhinitis	1	1	1		
**Tachycardia**	1	1	1		
**Upper respiratory tract infection**	1	1	1		
**TOTAL**		88	77	11	0

Adverse events in bold are those that were considered related to the malaria infection.

*Adverse events considered to be related to DSM265.

#Mild neck pain in one participant was considered related to the malaria infection, moderate neck pain in another participant was not considered related to study procedures/treatments.

Table S2: Malaria clinical score recorded for each participant at the time of first treatment with DSM265 and peak score recorded during the study

Participant ID	Clinical score at time of DSM265 treatment (Day 7)	Peak clinical score during study	Day/s of peak clinical score
R101	0	2	Day 5 PM, Day 10 PM
R102	0	2	Day 6 PM
R103	0	3	Day 8 PM
R104	3	4	Day 4 AM, Day 5 PM
R105	3	8	Day 8 PM
R106*	0	0	NA
R107	3	6	Day 9 PM
R108	0	3	Day 5 AM

The malaria clinical score served as a clinical indication of the severity of the induced malaria infection; 14 signs and symptoms commonly associated with malaria were graded using a 3-point scale (0=absent, 1=mild, 2=moderate, 3=severe) and the values were summed in order to generate an overall score (maximum possible score is 42).

*Participant R106 did not develop parasitemia at any point during the study.

NA = Not applicable

Table S3: Mosquito transmission assay results

Participant ID	Feeding rate (N mosquitoes fed/N total mosquitoes [%])		Mortality rate (N dead mosquitoes/N total mosquitoes [%])		Infection rate (N mosquitoes with oocysts/N mosquitoes tested [%])	
	**DFA**	**MFA**	**DFA**	**MFA**	**DFA**	**MFA**
**DAY 20**						
R101	33/33 (100%)	57/58 (98.3%)	1/33 (3%)	2/58 (3.4%)	0/30 (0%)	0/50 (0%)
R102	39/39 (100%)	64/68 (94.1%)	3/39 (7.7%)	8/68 (11.8%)	0/30 (0%)	0/50 (0%)
R103	37/37 (100%)	66/68 (97.1%)	0/37 (0%)	6/68 (8.8%)	0/30 (0%)	0/50 (0%)
R105	37/37 (100%)	57/63 (90.5%)	2/37 (5.4%)	3/63 (4.8%)	0/30 (0%)	0/50 (0%)
R106	37/37 (100%)	60/62 (96.8%)	2/37 (5.4%)	1/62 (1.6%)	0/30 (0%)	0/50 (0%)
R107	32/32 (100%)	64/64 (100%)	2/32 (6.3%)	1/64 (1.6%)	0/30 (0%)	0/50 (0%)
R108	38/38 (100%)	53/58 (91.4%)	4/38 (10.5%)	6/58 (10.3%)	0/30 (0%)	0/50 (0%)
**DAY 23**						
R101	NP	67/69 (97.1%)	NP	8/69 (11.6%)	NP	0/50 (0%)
R102	NP	64/66 (97%)	NP	6/66 (9.1%)	NP	0/50 (0%)
R103	NP	74/75 (98.7%)	NP	4/75 (5.3%)	NP	0/50 (0%)
R105	NP	81/82 (98.8%)	NP	8/82 (9.8%)	NP	0/50 (0%)
R106	NP	78/78 (100%)	NP	9/78 (11.5%)	NP	0/50 (0%)
R107	NP	65/65 (100%)	NP	3/65 (4.6%)	NP	0/50 (0%)
R108	NP	70/70 (100%)	NP	6/70 (8.6%)	NP	0/50 (0%)
**DAY 28**						
R101	35/35 (100%)	63/65 (96.9%)	5/35 (14.3%)	12/65 (18.5%)	0/30 (0%)	0/50 (0%)
R102	33/34 (97.1%)	65/66 (98.5%)	8/34 (23.5%)	10/66 (15.2%)	0/26 (0%)	0/50 (0%)
R103	36/38 (94.7%)	70/72 (97.2%)	5/38 (13.2%)	14/72 (19.4%)	0/30 (0%)	0/50 (0%)
R105	33/33 (100%)	78/82 (95.1%)	1/33 (3%)	24/82 (29.3%)	0/30 (0%)	0/50 (0%)
R106	33/34 (97.1%)	75/75 (100%)	5/34 (14.7%)	24/75 (32%)	0/29 (0%)	0/50 (0%)
R107	39/39 (100%)	62/64 (96.9%)	3/39 (7.7%)	11/64 (17.2%)	0/30 (0%)	0/50 (0%)
R108	39/39 (100%)	61/62 (98.4%)	9/39 (23.1%)	13/62 (21%)	0/30 (0%)	0/49 (0%)

DFA = direct feeding assay; DMFA = direct membrane feeding assay; NP = not performed

Supplementary text: Participant inclusion and exclusion criteria

INCLUSION CRITERIA

Participants eligible for inclusion in this study must fulfil **all** of the following criteria:

Demography

I01. Adults (male and non-pregnant, non-lactating female) participants between 18 and 55 years of age, inclusive who do not live alone (from Day 0 until at least the end of the anti-malarial drug treatment) and will be contactable and available for the duration of the trial and follow up period (maximum of 6 weeks).

I02. Body weight, minimum 50.0 kg, body mass index between 18.0 and 32.0 kg/m2, inclusive.

Health status

I03. Certified as healthy by a comprehensive clinical assessment (detailed medical history and complete physical examination).

I04. Normal vital signs after 5 minutes resting in supine position:

- 90 mmHg < systolic blood pressure (SBP) <140 mmHg,
- 50 mmHg < diastolic blood pressure (DBP) <90 mmHg,
- 40 bpm< heart rate (HR) <100 bpm.

I05. Normal standard 12-lead electrocardiogram (ECG) after 5 minutes resting in supine position, QTcF≤450 ms (males and females) with absence of second or third degree atrioventricular block or abnormal T wave morphology.

I06. Laboratory parameters within the normal range, unless the Investigator considers an abnormality to be clinically irrelevant for healthy participants enrolled in this clinical investigation. More specifically for serum creatinine, hepatic transaminase enzymes (aspartate aminotransferase, alanine aminotransferase), and total bilirubin (unless the Participant has documented Gilbert syndrome) should not exceed the acceptable range listed in Appendix 5 and haemoglobin must be equal or higher than the lower limit of the normal range.

I07. As there is the risk of adverse effects of the investigational drug (DSM265), and standard curative treatment (artemether-lumefantrine and primaquine) in pregnancy, it is important that any participants involved in this study do not get pregnant or get their female partners pregnant.

Female participants of childbearing potential (WOCBP) may be enrolled in the DSM265 cohorts but must have adequate contraception in place for the duration of the study and up to 60 days (9 weeks) after the last dose of DSM265, with adequate contraception defined as:

- Stable hormonal contraception (with an approved oral, transdermal or depot regimen) for at least 3 months prior to screening i.e. oral contraceptives, either combined or progestogen alone, hormonal implantable contraception, vaginal ring, contraceptive patches
- Intrauterine (IUD) device or system in place for at least 3 months prior to screening
- Male partner sterilization prior to the female participant's entry into the study, and this male is the sole partner for that participant

Abstinent female participants must agree to start a double method if they start a sexual relationship during the study and for up to 60 days (9 weeks) following the last dose of DSM265.

Male participants to be dosed with DSM265 must agree to use a double method of contraception including condom plus diaphragm or condom plus stable oral/transdermal/injectable hormonal contraceptive by female partner from at least 14 days prior to the time of the dose of the study drug through 120 days (17 weeks) after the last dose of DSM265.

Abstinent male participants must agree to start a double method if they start a sexual relationship during the study and for up to 120 days (17 weeks) weeks following the last dose of DSM265

Regulations

I09. Having given written informed consent prior to undertaking any study-related procedure.

EXCLUSION CRITERIA

Medical history and clinical status

E01. Any history of malaria or participation to a previous malaria challenge study
E02. Must not have travelled to or lived (>2 weeks) in a malaria-endemic country/area during the past 12 months or planned travel to a malaria-endemic country during the course of the study.
E03. Has evidence of increased cardiovascular disease risk (defined as >10%, 5 year risk when greater than 35 years of age) as determined by the Australian Absolute Cardiovascular Disease Risk Calculator (http://www.cvdcheck.org.au/). Risk factors include sex, age, systolic blood pressure (mm/Hg), smoking status, total and HDL cholesterol (mmol/L), and reported diabetes status.
E04. History of splenectomy.
E05. Presence or history of drug hypersensitivity, or allergic disease diagnosed and treated by a physician or history of a severe allergic reaction, anaphylaxis or convulsions following any vaccination or infusion.
E06. Presence of current or suspected serious chronic diseases such as cardiac or autoimmune disease (HIV or other immunodeficiencies), insulin-dependent and non-insulin dependent diabetes, progressive neurological disease, severe malnutrition, acute or progressive hepatic disease, acute or progressive renal disease, psoriasis, rheumatoid arthritis, asthma, epilepsy or obsessive compulsive disorder
E07. History of malignancy of any organ system (other than localized basal cell carcinoma of the skin or *in situ* cervical cancer), treated or untreated, within 5 years of screening, regardless of whether there is evidence of local recurrence or metastases
E08. Participants with history of schizophrenia, bi-polar disease, or other severe (disabling) chronic psychiatric diagnosis including depression or receiving psychiatric drugs or who has been hospitalized within the past 5 years prior to enrollment for psychiatric illness, history of suicide attempt or confinement for danger to self or others.
E09. Frequent headaches and/or migraine, recurrent nausea, and/or vomiting (more than twice a month)
E10. Presence of acute infectious disease or fever (e.g., sub-lingual temperature ≥ 38.5°C) within the five days prior to inoculation with malaria parasites.
E11. Evidence of acute illness within the four weeks before trial prior to screening that the Investigator deems may compromise subject safety.
E12. Significant inter-current disease of any type, in particular liver, renal, cardiac, pulmonary, neurologic, rheumatologic, or autoimmune disease by history, physical examination, and/or laboratory studies including urinalysis.
E13. Participant has a clinically significant disease or any condition or disease that might affect drug absorption, distribution or excretion, e.g. gastrectomy, diarrhoea.
E14. Participation in any investigational product study within the 12 weeks preceding the study.
E15. Blood donation, any volume, within 1 month before inclusion or participation in any research study involving blood sampling (more than 450 mL/ unit of blood), or blood donation to Red Cross (or other) blood bank during the 8 weeks preceding the reference drug dose in the study.
E16. Participant unwilling to defer blood donations to the ARCBS for 6 months.
E17. Medical requirement for intravenous immunoglobulin or blood transfusions.
E18. Participant who has ever received a blood transfusion.
E19. Symptomatic postural hypotension at screening, irrespective of the decrease in blood pressure, or asymptomatic postural hypotension defined as a decrease in systolic blood pressure ≥20 mmHg within 2-3 minutes when changing from supine to standing position.
E20. History or presence of alcohol abuse (alcohol consumption more than 40 g per day) or drug habituation, or any prior intravenous usage of an illicit substance.
E21. Smoking more than 5 cigarettes or equivalent per day and unable to stop smoking for the duration of the study.
E22. Ingestion of any poppy seeds within the 24 hours prior to the screening blood test (participants will be advised by phone not to consume any poppy seeds in this time period).
E23. Excessive consumption of beverages containing xanthine bases, including Red Bull, chocolate etc. more than 400 mg caffeine per day (equivalent to more than 4 cups per day)

Interfering substance
E24. Any medication (including St John's Wort) within 14 days before inclusion or within 5 times the elimination half-life (whichever is longer) of the medication
E25. Any vaccination within the last 28 days.
E26. Any corticosteroids, anti-inflammatory drugs, immunomodulators or anticoagulants. Any participant currently receiving or having previously received immunosuppressive therapy, including systemic steroids including adrenocorticotrophic hormone (ACTH) or inhaled steroids in dosages which are associated with hypothalamic-pituitary-adrenal axis suppression such as 1 mg/kg/day of prednisone or its equivalent or chronic use of inhaled high potency corticosteroids (budesonide 800 µg per day or fluticasone 750 µg).
E27. Any recent (< 6 weeks) or current systemic therapy with an antibiotic or drug with potential anti-malarial activity (i.e. chloroquine, piperaquine, benzodiazepine, flunarizine, fluoxetine, tetracycline, azithromycin, clindamycin, hydroxychloroquine, etc.)

General conditions
E28. Any participant who, in the judgment of the Investigator, is likely to be noncompliant during the study, or is unable to cooperate because of a language or mental deficit.
E29. Any participant in the exclusion period of a previous study according to applicable regulations.
E30. Any participant who lives alone (from Day 0 until at least the end of the anti-malarial drug treatment).
E31. Any participant who cannot be contacted in case of emergency for the duration of the trial and up to 2 weeks following end of study visit.
E32. Any participant who is the Investigator or any sub-investigator, research assistant, pharmacist, study coordinator, or other staff thereof, directly involved in conducting the study.
E33. Any participant without a good peripheral venous access.

Biological status
E34. Positive result on any of the following tests: hepatitis B surface (HBs Ag) antigen, anti-hepatitis B core antibodies (anti-HBc Ab), anti-hepatitis C virus (anti-HCV) antibodies, anti-human immunodeficiency virus 1 and 2 antibodies (anti-HIV1 and anti HIV2 Ab)
E35. Any drug listed in Table 2 (Drug Screening) in the urine drug screen unless there is an explanation acceptable to the medical investigator (e.g., the participant has stated in advance that they consumed a prescription or OTC product which contained the detected drug) and/or the Participant has a negative urine drug screen on retest by the pathology laboratory.

Specific to the study
E37. Cardiac/QT risk:
• Family history of sudden death or of congenital prolongation of the QTc interval or known congenital prolongation of the QTc interval or any clinical condition known to prolong the QTc interval.
• History of symptomatic cardiac arrhythmias or with clinically relevant bradycardia. Electrolyte disturbances, particularly hypokalaemia, hypocalcaemia, or hypomagnesaemia.
• Electrocardiogram (ECG) abnormalities in the standard 12-lead ECG (at screening) which in the opinion of the Investigator is clinically relevant or will interfere with the ECG analyses on study

E38. Known hypersensitivity to DSM265 or any of its excipients or 4-aminoquinolines, artemether or other artemisinin derivatives, lumefantrine, or other arylaminoalcohols.
E39. Known severe reaction to mosquito bites other than local itching and redness.
E40. Unwillingness to abstain from consumption of citrus (grapefruit, Seville orange, etc.) for ≥21 days prior to initiation of the study (inoculation; Day 0) and for the study duration.
E41. Unwillingness to abstain from consumption of quinine containing foods/beverages such as tonic water, lemon bitter, from inoculation (Day 0) to the end of the malaria treatment.
E42. Any history or presence of lactose intolerance.
E43. Use of prescription drugs, herbal supplements, within four weeks prior to administration of the study drug, and/or over-the-counter (OTC) medication, dietary supplements (including vitamins)

within two weeks prior to initial dosing (Note: diazepam interferes with the analysis of blood levels of DSM265 and thus should not have been used for at least 8 weeks prior to administration of the study drug). If needed (i.e. an incidental and limited need) paracetamol is acceptable up to 2 g/day.
Participants are requested to refrain from taking non-approved concomitant medication from recruitment until the conclusion of the study.
Participants who are excluded from participation on study days for any of the above reasons may be eligible to participate on a postponed schedule if the Investigator considers this appropriate.
